# Assessment of tuberculosis knowledge among Lesotho village health workers and utilisation of their tuberculosis services by the communities they serve

**DOI:** 10.4102/phcfm.v11i1.1944

**Published:** 2019-07-31

**Authors:** Maseabata M. Ramathebane, Tiisetso J. Makatjane, Regina M. Thetsane, Motselisi Mokhethi

**Affiliations:** 1 Faculty of Health Science, National University of Lesotho, Roma, Lesotho; 2 Faculty of Social Sciences, National University of Lesotho, Roma, Lesotho

**Keywords:** tuberculosis, inadequate knowledge, screening, treatment, village health workers, household

## Abstract

**Background:**

The role of village health workers (VHWs), among other roles is to educate communities about tuberculosis (TB), TB screening and its treatment. The knowledge of TB among VHWs is crucial because they will carry out their role at the community well and this will impact the overall outcome of TB treatment.

**Aim:**

The study is aimed at assessing the knowledge of TB among VHWs and households at the village level and the utilisation of VHWs’ TB services.

**Setting:**

The study took place in 19 health centres from 10 districts of Lesotho.

**Methods:**

The study used a cross-sectional descriptive design. Three study populations were interviewed, two at the household level (2040 households, 8295 individuals) and one at the clinic level (723 VHW).

**Results:**

Overall, TB knowledge among VHWs for the majority of clinics except two was inadequate (below mean of 31.5). The utilisation of VHWs’ TB services among community members was also low.

**Conclusion:**

Low utilisation of VHWs’ TB services by community members emanated from inadequate TB knowledge of VHWs. Regular refresher training among VHWs is recommended as the way forward in order to keep VHWs abreast with new TB developments.

## Introduction

Worldwide community health workers (CHWs) are expected to promote good health practices in the community through health education in matters that are of concern in the country.^1,2,3,4^ The most important matters of concern currently in Lesotho are a failure to meet Millennium Development Goals targets in HIV/AIDS, tuberculosis (TB), maternal and child mortality of which a village health worker (VHW) can play a vital role as one of the community members where she lives.^2,5,6^ For the VHWs to provide meaningful education to the community, he/she must have adequate information and knowledge about how to help to improve the health outcomes of TB^2,3,7^ hence the need to establish the level of knowledge of VHWs against the Ministry of Health’s VHWs training manual curriculum. It is equally important to establish if VHWs had access to refresher courses to periodically refresh their knowledge.

The criterion used for the selection of VHWs is stipulated in the Ministry of Health and Social Welfare (MOHSW) training manual and is in line with practices in other countries.^7,8^ According to the criterion for a person to be selected, he or she must be a full-time resident of the village with no other responsibilities. He or she must be elected by the village itself and be literate. He or she must be in good health and be an adult between the ages of 25–70 years. VHWs must have the following attributes: be a dedicated, trainable, and respected member of the community. He or she must be a person who maintains confidentiality and can work on a voluntary basis.^6,7^ In Lesotho, VHWs are seen as members of a primary health care team supervised by a nurse from the nearby clinic or health centre.^8,9^ He or she serves as a link between the community and primary health care facility.^7^

According to MOHSW (2011:9), VHWs operate through home visits, small group discussions and community gatherings.^7^ They must assist the village in developing and maintaining a safe water supply and sanitation;^9^ identify village health needs and facilitate the use of village health resources to meet these needs;^10^ assist the health centre team in controlling disease outbreaks and assist the chief with vital statistics (births and deaths registration); promote good nutrition and recognise, manage and organise follow-ups for under-nourished children;^9^ identify and provide initial treatment of diseases such as diarrhoea and vomiting;^9^ recognise, refer and organise follow-ups of TB patients;^11^ provide first aid and home-based care; participate in health centre meetings; keep patient records and report monthly activities to the health centre nurse; cooperate with development extension workers.^6,9^

An in-depth analysis of CHWs by Perry and Zullinger (p. 1)^5^ found that CHWs work under varied conditions and have a wide range of work environments and expectations.^5^ There are also disparities in the time taken to train VHWs where some have only a few days of training, while others have 6 months or more of training. In Lesotho, training of VHW is stipulated in the VHWs training manual by the Ministry of Health (MOH). For someone to qualify as a VHW, he or she must have completed 6 weeks of training using the MOH training manual.^6,7^ Competency of VHW has to be measured and improved through refresher courses for them to provide the expected services.^6,10^ Education for CHWs should include training on the more logistical aspects of their jobs, such as household entry, community sensitisation, data collection and recording, and relevant ethical issues mainly on how to maintain confidentiality.^6^

In 2003, Lesotho came up with an essential service package that specifies the role of VHWs, while in 2011 revitalisation of the health services strategy was established. These two strategies clearly specify the role of VHWs. Accordingly the health centre nurse has to provide supportive supervision to the VHWs and ensure that records are kept and available for inspection.^7,9^ There should be an organogram for the VHW which includes CHW supervisor and CHW coordinator, and the nurse who is part of the supervisory structure; this is highlighted by Rachlis et al.^6^ (p. 3) showing that there is a community health extension worker whose role is to supervise a group of CHWs.^6^ This allows the nurse to focus more on the clinical role and receive reports from the CHW coordinator who is based at the clinic, whose duties include collating information received from the CHW supervisors and submitting this to the nurse and clinic administrator.

Performance management should be carried out based on a standardised set of skills that respond with community needs.^10^ The programmes should have regular and continuous supervision and monitoring systems in place and supervision should be taught and undertaken in a participatory manner that ensures two-way flow of information.^10^ Other than assessing access to health care through VHWs,^9^ the researchers are not aware of any assessment study of TB knowledge of Lesotho’s VHWs. As the effectiveness of village health work is premised on the knowledge of VHWs, it is important that their knowledge is regularly evaluated. Other than providing feedback that could inform the training curriculum and the frequency of refresher courses, for Lesotho this is even more important as TB is among the top ten causes of death in the country. The long history of Basotho men working the South African mines and the association between TB and mine work could explain the high prevalence of TB in the country. An assessment of TB knowledge among VHWs, which has never been done before in Lesotho, is required if the country aims to improve TB treatment outcomes through community health care services. The study therefore has two objectives, namely to assess TB knowledge among the VHWs and to assess utilisation of VHWs’ TB services by the communities they serve.

## Methods

The study was conducted in Lesotho between 2016 and 2017 and covered all 10 districts in Lesotho. The study used a cross-sectional descriptive design. Three study populations were interviewed, two at the household level and one at the clinic level. The first sample was all household members whose information was provided by the head. The second sample was all present household members aged 15 years and above and a total of 1958 household members were interviewed. The third study population was made up of all VHWs serving in the selected clinics and 723 VHWs were interviewed.

The formula:

$$\text{n}=\left\{p(1-p){\left(1.96\right)}^{2}/d^{2}\right\},$$ [Eqn 1]

where *p* is the anticipated proportion (0.50) and *d* is the precision (0.05) required on either side of the proportion, estimated around the 95% confidence interval, was used to estimate the number of households within the catchment of the selected villages to be sampled. As the information on the number of community members receiving help from VHWs for HIV and/or TB treatment was not available, it was assumed, that 50% of the households within the community received help from the VHW, for purposes of sample size estimation. Furthermore, as the study utilises the multistage cluster sampling method, the sample size is multiplied by the design effect *(D = 2)*, plus a non-response rate of 10%. The estimated sample was 1200 households to the nearest 100. Lesotho’s population distribution by ecological zone was used to distribute households to be visited per clinic.

To maximise representativeness of the clinics in the sample, the health service area (HSA) concept was utilised where the country was divided into 17 catchment areas found around 17 hospitals in the country. Each hospital was responsible for supervising clinics within its catchment area. The distribution of health services was in accordance with the terrain of the country which has lowlands, foothills, mountains and the Senqu River. The 17 HSAs were used as clusters from which clinics were selected to represent each cluster. As there are clinics supervised or visited by Lesotho Flying Doctors Service (LFDS) and those supervised by the District Health Management Team (DHMT), clinics under LDFS and DHMT formed the 18th and 19th clusters.

Random numbers were used to select a clinic to represent each cluster. All data collection tools were uploaded on the Open Data Kit (ODK). All research assistants, supervisors and data managers signed a confidentiality form. Research assistants collected data using smart phones or tablets. Once the questionnaire was completed and checked for completeness, the information was submitted electronically to the server. If the research assistant was working in an area where there was no mobile network, completed files were queued and sent to the server immediately after the research assistant reached an area with a mobile network. Only blank forms were kept on the cell phone or tablet of the research assistant. In the event of the smart phone getting lost or stolen, the forms were still secure because one needs a password to access them. Only the data manager and the supervisors knew the passwords other than the research assistants. Data were collected between September and November 2016.

Eight questions were constructed to test for TB knowledge among VHWs. For each question, correct options were listed and the research assistant would tick the correct ones mentioned. The list of options was taken as is from the training manual. At the analysis stage, a code of 1 was given for the correctly mentioned options and a code of 0 was given for those not mentioned. To measure knowledge, a summary measure was computed by adding the responses of each question. In a question with four options, a summary score of 0 denoted that no correct option was mentioned, while a score of 4 would mean all correct options were mentioned. The options for each question ranged between 3 and 10. Overall knowledge was the summary of all the questions. To measure adequate knowledge, Nachega, Lehman, Hlatshwayo et al.’s (2005) definition of adequate knowledge was used.^12^ According to this definition, an individual must know at least 75% of the items used in the summary variable to qualify for adequate knowledge.

All data collection tools were translated from English into Sesotho. The Sesotho version of the tools was used for training both supervisors and research assistants. Tools were pretested to check if they were collecting required information. To avoid community complains with respect to why some households were visited while others were not visited, research assistants were to visit each household within the selected village even if that meant going beyond the number of households for the clinic. On the other hand, if the households of the selected villages fell short of the target, even if by five households, another village had to be included in the sample and all households in the village were to be visited not just the additional five to meet the target. This approach has resulted in almost double (2140 instead of 1200) the number of households visited.

At the household level, the head was asked to report on all household members if they had ever been diagnosed with TB. For those who had ever been diagnosed, the head was to report if the VHW helped the member with TB treatment. For household members aged 15 years and above, five questions were asked, namely about how TB is spread from one person to another the causes of TB, whether TB can be cured, the signs that someone could be suffering from TB and whether the respondent was advised by the VHW to do a screening for TB. These five questions were addressed to respondents who acknowledged knowledge of TB. For the household level questions, the options for questions included known myths about TB.

As the assessment of knowledge was based on the information contained in the training manual, questions were formulated using what VHWs had been taught during training. The exceptions were questions on the profile of VHWs such as age and the year of training. As VHWs would teach what they had been taught during training, questions for members of the community were also based on the training curriculum of VHWs. As this was the first assessment for the country, we had nothing with which to benchmark the tools.

Data were analysed in November 2016 using Statistical Package for Social Scientists (SPSS) version 20. Goodwill permission to conduct the study was obtained from the 19 selected clinics. All the study participants gave their written informed consent. Key information pertaining to treatment of TB comparing the performance of clinics was prepared and taken to the clinics to validate with all VHWs participating in the study.

### Ethical considerations

Ethical approval was obtained from the National Ethics and Review Board of the Ministry of Health – registration number 188-2016 (Reference number: ID73-2016).

## Results

### Characteristics of household members

In total 2040 households were visited during the survey with a population of 8295 individuals. Regarding the profile of household members, more than a third (37%) of the household members are children while grandchildren constitute 17%. More than half (52%) were females and 48% were males. Forty-two per cent of the household members were aged less than 20 years, while 13% were aged 60 years and above. About a fifth (17%) of the members were absent from the household, while 82% were present. More than half (54%) of the household members had finished their primary level education compared to a quarter (25%) who had done secondary education or better. Less than 40% (38%) of the household members were never married compared to 47% and 16% of them either currently married or previously married, respectively. Table 1 presents the profile of household members.

**TABLE 1:** Characteristics of the household population.

Characteristic	Category	%	*N*
Relationship to head	Head	23.8	1970
	Spouse	12.4	1031
	Child	37.0	3068
	Son- or daughter-in-law	1.9	156
	Grandchild or great grandchild	16.8	1391
	Other relative	6.4	535
	Other person not related	1.6	132
	No response	0.1	12
Sex	Male	47.8	3963
	Female	52.2	4326
	No response	0.0	6
Age	00–10	18.8	1559
	10–19	22.9	1902
	20–29	16.1	1338
	30–59	24.7	2046
	60+	13.2	1095
	Age not stated	4.3	355
Residential status	Present	81.8	6786
	Visitor	0.6	49
	Member elsewhere in Lesotho	9.2	761
	Member outside Lesotho	8.2	681
	Do not know	0.1	6
	No response	0.1	12
Marital status	Never married	37.8	2162
	Currently married	46.7	2669
	Previously married	15.5	884
Level of education	Pre-school	3.3	273
	Primary	54.1	4489
	Secondary and above	25.0	2077
	Other	0.2	14
	Do not know	0.5	44
	Aged less than 5 or no response	16.9	1398

### Characteristics of village health workers

More than 700 (723) VHWs responded to the VHW questionnaire. The age distribution of VHWs is bell shaped. It increases from a low of 18% for those aged less than 40 years and reaches a peak of 28% for those aged between 50 and 59 years. Almost all VHWs are females (93%), while the majority (64%) are currently married and a third (33%) were previously married. More than two-thirds (77%) of the VHWs had completed primary education and a fifth had completed secondary education or higher. Almost all VHWs (94.6%) reported that they were trained by the MOH and were mostly trained after 1999 while refresher courses were concentrated between 2010 and 2016. Table 2 shows the profile of VHW.

**TABLE 2:** Profile of village health workers.

Characteristic	Category	%	*N*
Sex	Male	6.6	48
	Female	93.2	674
	No response	0.1	1
Age	< 40	18.0	130
	40–49	25.4	184
	50–59	28.1	203
	60–69	22.8	165
	70+	5.2	38
	No response	0.4	3
Marital status	Never married	2.2	16
	Currently married	64.4	467
	Previously married	33.2	241
	No response	0.1	1
Education	No education	1.2	9
	Primary	77.4	561
	Secondary or higher	21.2	154
	No response	0.1	1
Who trained you?	Ministry of health	94.6	684
	Partner In Health, world vision, global health	2.9	21
	No training	2.5	18
Year of training	Before 1990	9.3	67
	1990–1999	14.7	106
	2000–2009	28.6	207
	2010–2016	38.6	279
	Do not remember	6.4	46
	No training	2.5	18
Year of refresher course	Before 2000	1.8	12
	2000–2009	5.8	42
	2010–2016	58.8	420
	Do not remember	20.5	147
	No refresher course	11.6	84
	No training	2.5	18

PIH, partners in health.

### Tuberculosis knowledge among household members

**FIGURE 1:**
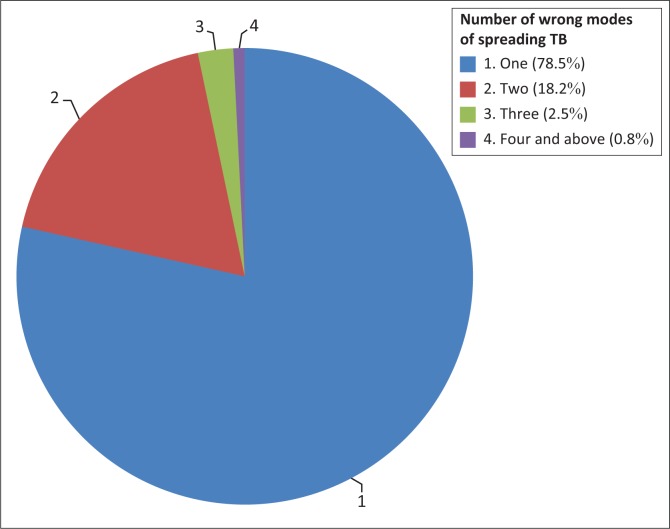
Number of wrong modes of spreading tuberculosis mentioned (%).

The majority (87.9%) of the household members aged 15 years and above who had ever heard of TB knew that TB is spread through the air when coughing or sneezing. Wrong modes of spreading TB such as touching someone who has TB, sharing food or utensils with someone who has TB or unprotected sex as well as mosquito bites were also mentioned by the remaining 12% of the respondents. As shown in Figure 1, 78.5% mentioned one wrong mode of spreading TB, while 18 respondents mentioned two wrong modes of spreading TB. Less than 5% mentioned or identified at least three wrong modes of spreading TB. Touching someone who has TB or sharing utensils with someone who has TB was mentioned by 6% of the respondents, while sharing food with someone who has TB, sexual contact or mosquito bites was mentioned by less than 3%. Figure 1 shows the number and percentage of wrong modes of spreading TB mentioned.

**FIGURE 2:**
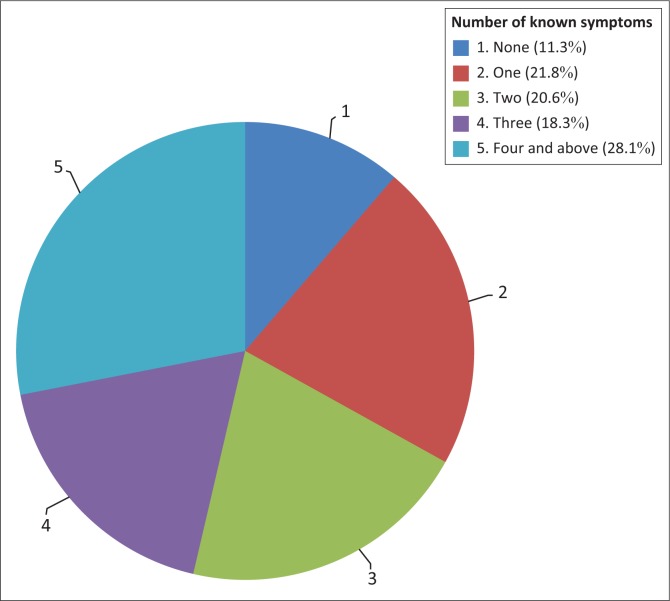
Number of known symptoms that someone has tuberculosis (%).

Concerning the knowledge of symptoms for someone for who has TB, 28.1% of the respondents knew at least 4 of the 9 symptoms. One in five knew between 1 to 3 symptoms, while 11.3% knew none of the symptoms. Coughing continuously for 2 weeks was the most known symptom mentioned by 52% of the respondents followed by sweating at night mentioned by 48.7%. Coughing with sputum, loss of appetite and weight loss were mentioned by between 30.4% and 38.9%. Fever, chest pains and tiredness or fatigue were mentioned by between 10.5% and 14%, while blood in sputum was mentioned by 20.8% (Figure 2). Figure 2 shows the number and percentage of known symptoms that someone has TB.

**FIGURE 3:**
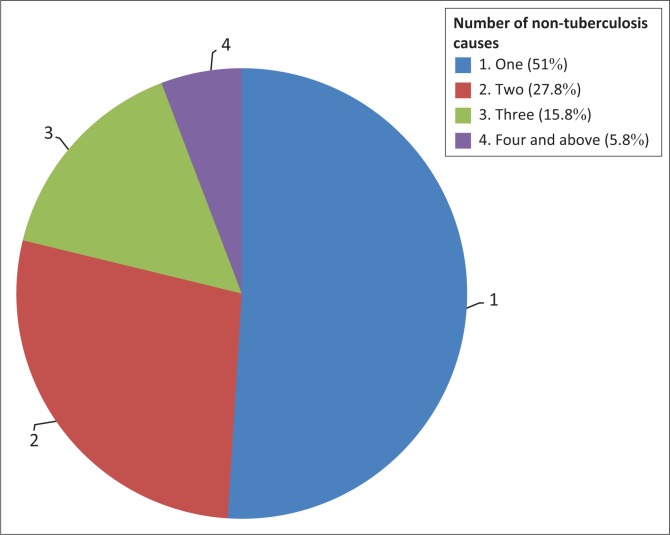
Number of non-tuberculosis causes mentioned (%).

With respect to what causes TB, 41% of the respondents knew that TB is caused by microbes or germs or bacteria found in the air. More than half of the respondents were of the view that TB is caused by other causes including inheritance from parents, smoking, alcohol drinking, exposure to cold temperatures and dust. Smoking (32.5%), dust (29.7%) and alcohol consumption (15.8%) to a larger extent were the major culprits believed to cause TB. The other non-causes of TB were mentioned by between 2.9% and 8.7%. However, only less than 5% of the respondents who associated causes of TB with wrong modes associated TB with at least 4 wrong causes, while the majority (51%) associated it with one wrong cause (Figure 3). Figure 3 depicts the number and percentage of non-TB causes mentioned.

### Tuberculosis knowledge among village health workers

Generally speaking there was inadequate TB knowledge among VHWs. There were, however, disparities across the indicators. For knowledge about signs that someone has TB and what can be observed after completion of TB treatment, the shortfall between the observed and expected mean was 25% and 33% for the knowledge of side effects of TB drugs. The highest disparity was for the knowledge of side effects of multi drug resistance (MDR) TB with a short fall of 67%. For the remaining measures the shortfall was between 50% and 57%. Table 3 shows the mean knowledge of TB among VHWs. For the overall knowledge, the shortfall between the observed and the expected is more than 50% (53.9%). It is noteworthy that at the clinic level, VHWs of two clinics had adequate knowledge with a mean of 32.96 and 37.00. Table 3 presents the knowledge of TB among VHWs.

**TABLE 3:** Knowledge of tuberculosis among village health workers.

Knowledge summary measure of:	Observed mean	Expected mean	*N*
Signs that someone has TB	3.28	4.50	6
Predisposing factors for TB infection	2.30	4.50	6
Ways of preventing the spread of TB infection	2.22	3.00	4
Some side effects of TB drugs	2.78	7.50	10
Some side effects of MDR-TB treatment	2.27	6.75	9
Importance of TB treatment	1.71	2.23	3
What may be observed after completion of TB treatment	2.41	3.00	4
Overall knowledge	14.55	31.50	42

Note: *N* denotes number of responses used to compute the summary measure.

Expected mean is based on Nachega et al.’s definition of adequate knowledge.^12^

TB, tuberculosis.

### Utilisation of village health workers’ tuberculosis services

**FIGURE 4:**
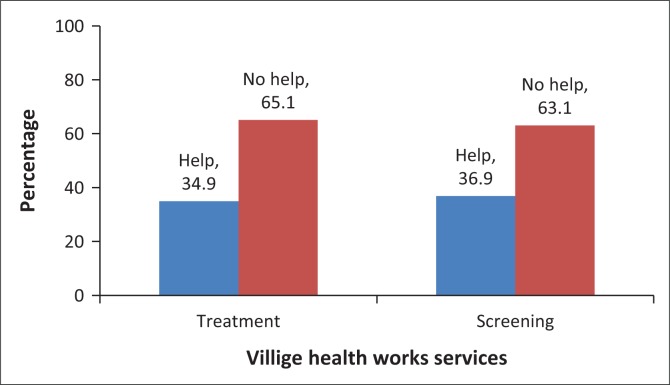
Percentage of household members who received help from village health workers for tuberculosis treatment or screening.

Responding to who helped the household members to be on TB treatment, the household head reported that a third (34.9%) of those diagnosed with TB were helped by the VHW. Among the household members aged 15 years and above, more than a third (36.9%) reported that they were advised by the VHW to go for TB screening (see Figure 4). One reported about advice to go for TB screening (self-reported), while the other reported about help to get TB treatment (household head). However, both reports were similar in terms of the utilisation of services of the VHW which is estimated at around 33%. Figure 4 presents the utilisation of the VHWs’ TB services by community members and the percentage of household members who received help from VHWs for TB treatment or screening.

## Discussion

Generally speaking, there is inadequate TB knowledge among Lesotho’s VHWs. The disparity between observed mean knowledge and expected knowledge is huge. The low utilisation of the VHWs’ TB services among community members was not surprising because of inadequate TB knowledge among VHWs. Understandably, community members served by a VHW with inadequate knowledge are less likely to utilise VHWs’ TB services. This finding confirmed what was suggested by the literature, namely that adequate knowledge among VHWs would translate into high or better utilisation of VHWs’ services.^13,14,15^

The information on the profile of VHWs (Table 2) does not offer any explanation about the prevailing inadequate knowledge among VHWs. According to Table 2, close to 6 in 10 (58.8%) VHWs had had refresher training recently, between 2010 and 2016, ruling out the lack of a refresher course as the main factor behind inadequate knowledge. It is important to further investigate factors behind inadequate knowledge among VHWs, more so because all VHWs (94.6%) except one or two claimed to have been trained by the MOH. The practices of the two clinics with adequate knowledge might share some light about why VHWs from other clinics are not performing accordingly.

## Limitations of the study

What emerged during the course of conducting the study was that clinics operate differently and, during the planning stages of the study, no adequate steps were taken to control for some of the differences. One major difference was the presence or absence of a VHW coordinator although this was in less than five clinics. Unlike in circumstances where there is no coordinator, the coordinator has enough time to attend to VHWs’ problems and concerns, thus creating an enabling environment. One other difference was that in some clinics, partners in health provided additional remuneration to VHWs which boosted their morale. Such dynamics ought to have been taken into consideration if the study was not the first of its kind. Future studies should take the differences in the way clinics function into consideration when assessing factors associated with inadequate TB knowledge.

## Conclusion and recommendations

Inadequate TB knowledge among Lesotho’s VHWs and the communities they serve is the cause for concern. Improved TB treatment outcomes are not likely to be realised in the near future before inadequate TB knowledge among VHWs is addressed through rigorous training. It might also be in order to investigate the perceptions of community members on the role of the VHWs in their villages. This is particularly so because it is common knowledge that patients or their relatives, in particular people living with HIV, get nothing when VHWs are compensated for reporting on their status. As Basotho respect their local authorities and take their word to be final, local authorities need some orientation on the work of the VHW so that both the village chief and the VHW can come to an understanding.
